# Experimental data on the compressive and flexural strength of lateritic paving tiles compounded with pulverized cow bone

**DOI:** 10.1016/j.dib.2020.106511

**Published:** 2020-11-06

**Authors:** Peter Omoniyi, Idehai Ohijeagbon, Jacob Aweda, Olatunji Abolusoro, Esther Akinlabi

**Affiliations:** a Department of Mechanical Engineering Science, University of Johannesburg, South Africa; b Department of Mechanical Engineering, University of Ilorin, Nigeria; c Pan African University for Life and Earth Sciences Institute (PAULESI), Ibadan, Nigeria

**Keywords:** Casting, Compressive strength, Flexural strength, Laterite, Sharp sand, Pulverized cow bones

## Abstract

This article presents the data of the bulk density, compressive strength and flexural strength of lateritic paving tiles compounded with pulverized cow bones (PCB) as reinforcement, the data set are presented in three categories. Category A involves the mixture of laterite and PCB, category B involves the mixture of sharp sand and PCB, lastly, category C involves the mixture of laterite, sharp sand and PCB. The paving tiles were made using the casting method in a 200 × 100 × 60 mm mould, using 20, 15, 10% wt. Portland cement as a binder and cured for 28 days in a curing tank. The data provided will give useful information for predicting the mechanical properties of paving tiles at different PCB constituent percentage.

## Specifications Table

**Table:** 

Subject	Mechanical Engineering, Civil and Structural Engineering
Specific subject area	Waste Management, recycling, Construction Materials
Type of data	Table Figure Chart
How data were acquired	Casting of paving tiles compounded with pulverized cow bones in the laboratory and carrying out bulk density test, compressive test and flexural strength tests
Data format	Raw
Parameters for data collection	Samples produced were cured in curing tank at room temperature for 28 days.
Description of data collection	Data were collected from the readings of the universal testing machine (UTM) FS50AT
Data source location	Institution: University of Ilorin City/Town/Region: Ilorin Country: Nigeria Latitude and longitude (and GPS coordinates, if possible) for collected samples/data: 8.4928°N, 4.5962°E
Data accessibility	Data are as presented in this article.
Related research article	J. O. Aweda, P. O. Omoniyi, I. O. Ohijeagbon, Suitability of Pulverized Cow Bones as a Paving Tile Constituent, Int. Conf. Eng. Sustainable World 2018. https://doi:10.1088/1757-899X/413/1/012046

## Value of the Data

• The data set gives an opportunity for further improvement in the knowledge base and applications of the suitability of PCB as a paving tile constituent.
• The building and road sector will hugely benefit from the data
• The data set will be helpful in building an empirical model for the prediction of the mechanical properties of lateritic paving tiles
• The test data will also allow further investigation into the mechanical behaviour of the lateritic paving tiles.
• The data will provide requisite information and guide in the planning and designing of sustainable and affordable paving tiles in the construction sectors.

## 1 Data description

The data provided contains the information on the bulk density, compressive strength and flexural strength of PCB reinforced lateritic tiles, with 20, 15 and 10% Portland cement addition as a binder.

**Fig 1:**
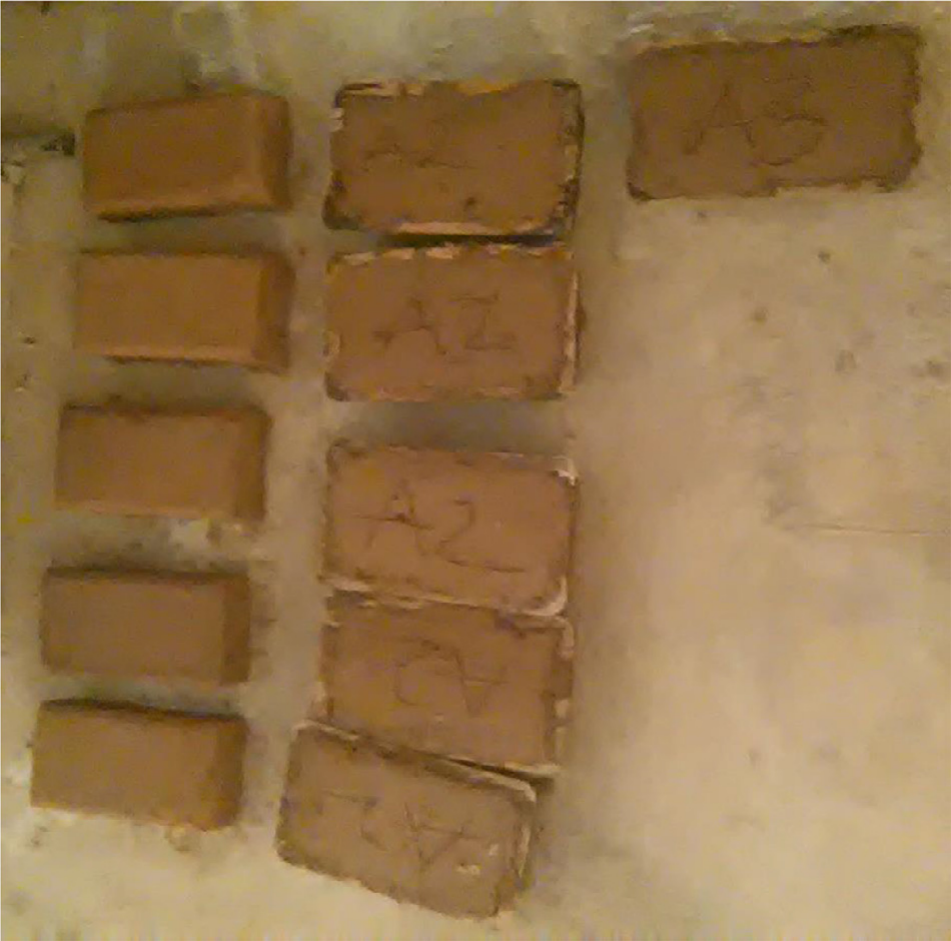
Paving tiles samples.

**Fig 2:**
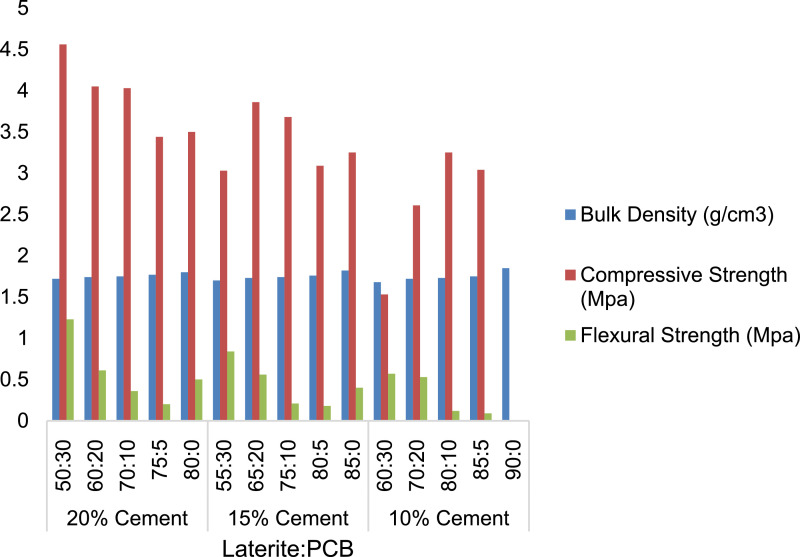
Engineering properties of paving tiles made with laterite and PCB.

**Fig 3:**
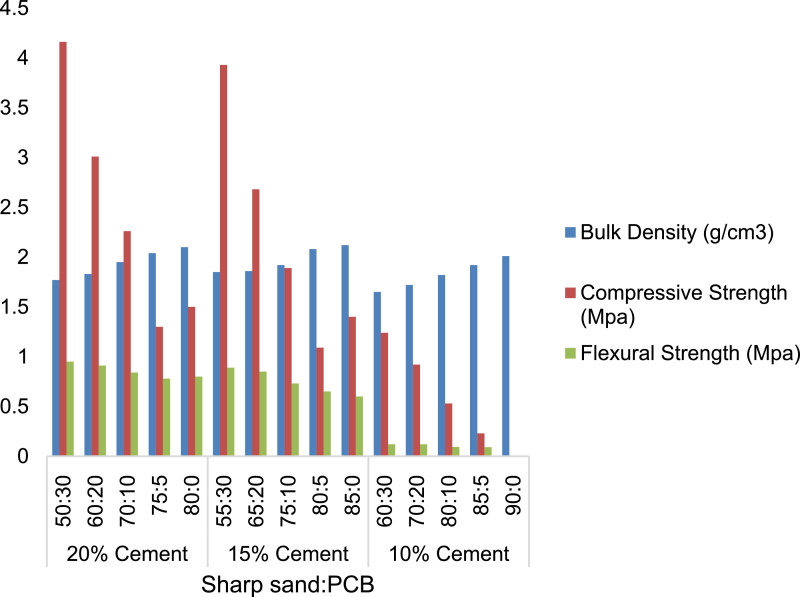
Engineering properties of paving tiles made with sharp sand and PCB.

**Fig. 4:**
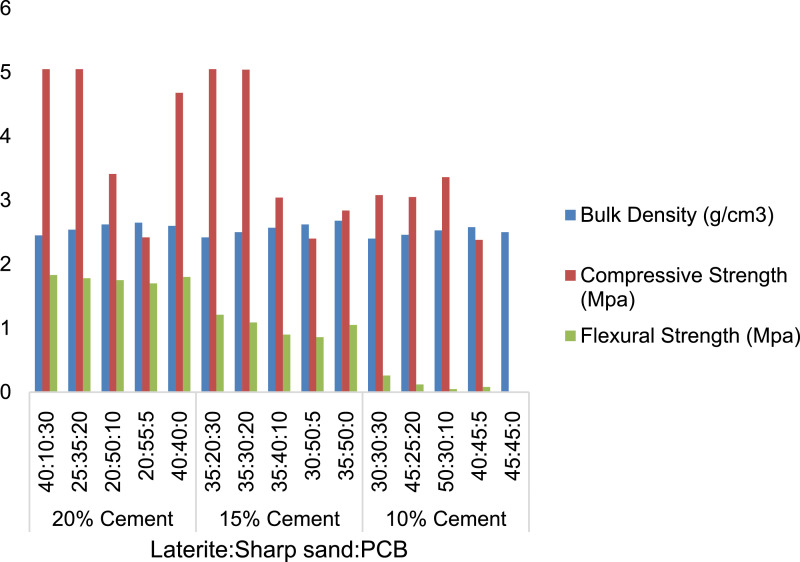
Engineering properties of paving tiles made with laterite, sharp sand and PCB.

Table 1, Table 2, Table 3 shows the mechanical properties of category A paving tiles with 20, 15 and 10% cement addition respectively and mixture of PCB with laterite, Table 4, Table 5, Table 6 shows the mechanical properties of category B paving tiles, with 20, 15 and 10% cement addition respectively and mixture of PCB with sharp sand. Table 7, Table 8, Table 9 shows the mechanical properties of category C paving tiles with 20, 15, 10% cement addition respectively and mixture of PCB, sharp sand and laterite. Fig. 1 shows a typical lateritic paving tile produced. Finally, Figs. 2–4 shows the performance of the paving tiles at different cement composition.

**Table 1:** Physical and Mechanical Properties of Experimental Lateritic Paving Tiles with 20% Cement Content and Laterite.

Sample Composition					
Pulverized Cow Bones(%)	Laterite (%)	Cement (%)	Bulk density (g/cm^3^)	Compressive Strength (MPa)	Flexural Strength (MPa)
30	50	20	1.72	4.56	1.23
20	60	20	1.74	4.05	0.53
10	70	20	1.75	4.03	0.36
5	75	20	1.77	3.44	0.20
0	80	20	1.80	3.50	0.50

**Table 2:** Physical and Mechanical Properties of Experimental Lateritic Paving Tiles with 15% Cement Content and Laterite.

Sample Composition					
Pulverized Cow Bones (%)	Laterite (%)	Cement (%)	Bulk density (g/cm^3^)	Compressive Strength (MPa)	Flexural Strength (MPa)
30	55	15	1.70	3.03	0.84
20	65	15	1.73	3.86	0.54
10	75	15	1.74	3.68	0.21
5	80	15	1.76	3.09	0.18
0	85	15	1.82	3.25	0.40

**Table 3:** Physical and Mechanical Properties of Experimental Lateritic Paving Tiles with 10% Cement Content and Laterite.

Sample Composition					
Pulverized Cow Bones (%)	Laterite (%)	Cement (%)	Bulk density (g/cm^3^)	Compressive Strength (MPa)	Flexural Strength (MPa)
30	60	10	1.68	1.53	0.57
20	70	10	1.72	2.61	0.56
10	80	10	1.73	3.23	0.12
5	85	10	1.75	3.04	0.09
0	90	10	1.85	3.05	0.30

**Table 4:** Physical and Mechanical Properties of Experimental Lateritic Paving Tiles with 20% Cement Content and Sharp Sand.

Sample Composition					
Pulverized Cow Bones (%)	Sharp Sand (%)	Cement (%)	Bulk density (g/cm^3^)	Compressive Strength (MPa)	Flexural Strength (MPa)
30	50	20	1.77	4.16	0.95
20	60	20	1.83	3.01	0.91
10	70	20	1.95	2.26	0.84
5	75	20	2.04	1.30	0.78
0	80	20	2.10	1.50	0.80

**Table 5:** Physical and Mechanical Properties of Experimental Lateritic Paving Tiles with 15% Cement Content and Sharp Sand.

Sample Composition					
Pulverized Cow Bones (%)	Sharp Sand (%)	Cement (%)	Bulk density (g/cm^3^)	Compressive Strength (MPa)	Flexural Strength (MPa)
30	55	15	1.85	3.93	0.89
20	65	15	1.86	2.68	0.85
10	75	15	1.92	1.89	0.73
5	80	15	2.08	1.09	0.65
0	85	15	2.12	1.40	0.60

**Table 6:** Physical and Mechanical Properties of Experimental Lateritic Paving Tiles with 10% Cement Content and Sharp Sand.

Sample Composition					
Pulverized Cow Bones (%)	Sharp Sand (%)	Cement (%)	Bulk density (g/cm^3^)	Compressive Strength (MPa)	Flexural Strength (MPa)
30	60	10	1.65	1.24	0.12
20	70	10	1.72	0.92	0.12
10	80	10	1.82	0.53	0.09
5	85	10	1.92	0.23	0.09
0	90	10	2.01	0.22	0.10

**Table 7:** Physical and Mechanical Properties of Experimental Lateritic Paving Tiles with 20% Cement Content, Sharp Sand and Laterite.

Sample Composition						
Pulverized Cow Bones (%)	Sharp Sand (%)	Laterite (%)	Cement (%)	Bulk density (g/cm^3^)	Compressive Strength (MPa)	Flexural Strength (MPa)
30	10	40	20	2.45	5.05	1.83
20	35	25	20	2.54	5.05	1.79
10	50	20	20	2.62	3.41	1.75
5	55	20	20	2.65	2.42	1.70
0	40	40	20	2.60	4.68	1.80

**Table 8:** Physical and Mechanical Properties of Experimental Lateritic Paving Tiles with 15% Cement Content, Sharp Sand and Laterite.

Sample Composition						
Pulverized Cow Bones (%)	Sharp Sand (%)	Laterite (%)	Cement (%)	Bulk density (g/cm^3^)	Compressive Strength (MPa)	Flexural Strength (MPa)
30	20	35	15	2.42	5.05	1.21
20	30	35	15	2.50	5.04	1.09
10	40	35	15	2.57	3.04	0.90
5	50	30	15	2.62	2.40	0.86
0	50	35	15	2.68	2.84	1.05

**Table 9:** Physical and Mechanical Properties of Experimental Lateritic Paving Tiles with 10% Cement Content, Sharp Sand and Laterite.

Sample Composition						
Pulverized Cow Bones (%)	Sharp Sand (%)	Laterite (%)	Cement (%)	Bulk density (g/cm^3^)	Compressive Strength (MPa)	Flexural Strength (MPa)
30	30	30	10	2.40	3.08	0.26
20	25	45	10	2.46	3.05	0.12
10	30	50	10	2.53	3.36	0.05
5	45	40	10	2.58	2.38	0.08
0	45	45	10	2.50	2.34	0.10

## 2 Experimental Design, Materials and Methods

The pulverized cow bones (PCB) used in this research study consist of waste femurs, scapulars and ribs of cow procured from an abattoir in Ilorin, Nigeria. They were washed and sun-dried for 4 weeks, to reduce the moisture content and eliminate the organic matter in the marrows of the bones. Thereafter, they were crushed and pulverized using a laboratory ball mill. The properties of other materials such as lateritic soil and sharp sand used are same as used in [1] and were carried out in accordance with the appropriate standards of American society for test and materials standard (ASTM). The pulverized cow bones were mixed with the lateritic soil and sharp sand. Cement satisfying the requirements of ASTM C150/C150M [2] was used as binder in various proportions.

Percentage composition of cement was chosen, based on literature review, where researchers [3,4] have observed that percentage addition of cement above 20%, will not further improve the mechanical properties of tiles and will be of less economic importance. Therefore, the mixing ratios are indicated in Table 1, Table 2, Table 3, Table 4, Table 5, Table 6, Table 7, Table 8, Table 9.

Each paving tile was produced by adopting the method used by Aweda et al., [1] and ASTM C685/C685M [5]. universal tensile machine FS50AT was used in carrying out the compressive and flexural strength of the paving tiles, following ASTM standards [6,7] respectively. Bulk density was carried out in accordance with ASTM C948 [8]. The paving tiles were curred in a curing tank for 28 days.

## Ethics Statement

All experiments complied with the ARRIVE guidelines and were carried out in accordance with the U.K. Animals (Scientific Procedures) Act, 1986 and associated guidelines, EU Directive 2010/63/EU for animal experiments.

## Declaration of Competing Interest

None declared.

## Appendix Supplementary Materials

Image, application 1
